# Seasonal changes in the essential oils of *Aloysia oblanceolata* Moldenke 

**DOI:** 10.3389/fchem.2025.1564404

**Published:** 2025-06-20

**Authors:** Paulo Vinicius L. Santos, Mateus Machado Lima, Flavia Cristina A. Lucas, Joyce Kelly do R. da Silva, José Guilherme S. Maia, Pablo Luis B. Figueiredo

**Affiliations:** ^1^ Programa de Pós-Graduação em Ciências Farmacêuticas, Instituto de Ciências da Saúde, Universidade Federal do Pará, Belém, Brazil; ^2^ Laboratório de Química dos Produtos Naturais, Universidade do Estado Pará, Belém, Brazil; ^3^ Programa de Pós-Graduação em Ciências Ambientais, Universidade do Estado do Pará, Belém, Brazil; ^4^ Programa de Pós-Graduação em Biotecnologia, Instituto de Ciências Biológicas, Universidade Federal do Pará, Belém, Brazil

**Keywords:** lavender, Verbenaceae, mono- and sesquiterpenes, essential oil composition, environmental factors

## Abstract

**Introduction:**

Aromatic plant species that produce essential oils contain bioactive compounds and medicinal properties, and are thus used in traditional medicine. This study aims to analyze the seasonal variation in *Aloysia oblanceolata* Moldenke's essential oil's chemical composition in the Amazon region.

**Methods:**

The botanical material was collected monthly over 1 year; the leaf essential oils were extracted by hydrodistillation, and their chemical composition was analyzed through gas chromatography–mass spectrometry (GC-MS). Local climatic parameters, such as insolation, temperature, relative humidity, and precipitation, were monitored. Multivariate statistical analyses were performed using hierarchical cluster analysis (HCA) and principal component analysis (PCA).

**Results and discussion:**

The AoEO yields ranged from 3.4% (December 2022 and February 2023) to 5.3% (August and September 2023), with an average of 4.3% ± 0.7%. Essential oil yields did not show a significant difference (p > 0.05) between the dry (4.7% ± 0.7%) and rainy (4.1% ± 0.6%) periods. GC and GC-MS identified 38 chemical constituents in the essential oils, comprising approximately 94.6%–97.1% of the oils analyzed in the seasonal variation study over the 12-month period. The chemical constituents that significantly correlated with climatic factors were *trans*-pinocamphone, β-pinene, and *E*-caryophyllene. Group I was statistically different from Groups II and III in terms of *trans*-pinocarvyl acetate content (I = 7.0 ± 0.5%; II = 9.1 ± 0.3%; III = 8.2 ± 0.6%). Group II differed from the other groups in terms of its β-pinene (I = 4.3 ± 0.5%; II = 5.2 ± 0.3%; III = 1.6 ± 0.9%), *trans*-pinocamphone (I = 13.1 ± 1.2%; II = 15.9 ± 0.4%; III = 12.5 ± 2.1%), and *E*-caryophyllene contents (I = 6.9 ± 0.7%; II = 4.7 ± 0.1%; III = 7.9 ± 1.6%). Furthermore, Group III differed from the other groups in terms of β-pinene content (I = 4.3 ± 0.5%; II = 5.2 ± 0.3%; III = 1.6 ± 0.9%). These results indicate seasonal variations in the chemical composition of the essential oils, possibly influenced by environmental factors and plant development.

## 1 Introduction

The Verbenaceae family, classified in the Lantaneae tribe, harbors a wide diversity of shrubs, trees, and herbs (O’Leary et al., 2016a). These plants have inflorescences and perennial leaves generally arranged oppositely (Romero et al., 2002). Taxa belonging to this family are used in traditional medicine due to the presence of bioactive compounds and their medicinal properties, including antioxidant and anti-inflammatory actions (Mohammadhosseini et al., 2022).

Aromatic plant species are used in traditional medicine due to the presence of bioactive compounds and their medicinal properties (Zeni et al., 2011; Mohammadhosseini et al., 2022). The *Aloysia* genus (Verbenaceae) is native to Brazil and originates from South America, especially in Argentina, Bolivia, Brazil, and Chile. These regions are known for harboring the greatest diversity of *Aloysia* species, with 28 species and 6 varieties, 12 of which are found in Brazil (Benovit et al., 2015; Mohammadhosseini et al., 2022).

*Aloysia oblanceolata* Moldenke (syn. *Aloysia gratissima* var. Oblanceolata Moldenke), popularly known as “Alfazema” and “Vassourinha Doce,” is a shrub with fasciculated leaves arranged oppositely along the branches, producing white inflorescences composed of flowers grouped in the same structure (O’Leary et al., 2016a). This species is native to South America, occurring in Paraguay, Bolivia, and Brazil. In Brazil, *Aloysia oblanceolata* is mainly found in the states of Rio Grande do Sul and Paraná (Marx et al., 2010).

*A. oblanceolata* is used in traditional medicine due to its sedative properties, with central nervous system depressant activity (Gressler et al., 2014; Benovit et al., 2015). The essential oils from *A. oblanceolata* have received much attention due to their antibacterial, antifungal, and antimycotic properties (Souza and Wiest, 2007).

There is significant scientific interest in researching plant-derived compounds associated with folk medicine knowledge. In this context, essential oils represent an important source of natural substances, since their active constituents often exhibit many pharmacological properties (de Cássia da Silveira e Sá et al., 2014). In addition, natural products are active against microorganisms, such as fungi, viruses, and bacteria, that are responsible for several infectious diseases (Ud-Daula et al., 2016; Alijar Souza et al., 2022).

Several environmental factors can significantly impact the chemical composition of essential oils produced by plants since these substances are primarily influenced by conditions external to the plant organism. Among the most relevant aspects that contribute to this variation are the incidence of solar radiation, which directly affects photosynthesis and, consequently, the synthesis of secondary metabolites; rainfall levels, which regulate water availability and osmotic balance; and variations in temperature and pressure, which can interfere with the processes responsible for the biosynthesis of volatile compounds (Gobbo-Neto and Lopes, 2007; Jerônimo et al., 2024).

These complex interactions between biotic and abiotic factors highlight the importance of understanding environmental and regional dynamics in the study of the production and quality of essential oils. Given this perspective, this study aimed to analyze the seasonal variation in the chemical composition of the essential oils from *Aloysia oblanceolata*.

## 2 Methodology

### 2.1 Plant material and climate data

Leaves (150 g) of a cultivated *A. oblanceolata* were collected from a home garden in the city of Garrafão do Norte, Pará state, Brazil (coordinates: 1°56′22.7382″S/47°3′3.17772W). Mature leaves from a single specimen were sampled monthly on the third day of every month, at 9 a.m., from November 2022 to August 2023. To ensure an unequivocal taxonomic identification, three samples were sent for independent identification, and vouchers were incorporated into the Herbarium of the Museu Paraense Emílio Goeldi (MG246092), the Herbarium of the Universidade do Estado do Pará (MFS10607), and the Herbarium of Embrapa Amazônia Oriental (IAN202959). The specimen was collected in accordance with Brazilian laws regarding biodiversity protection (Sisgen ACA3523).

The data on climatic parameters (insolation, relative humidity, and rainfall) were obtained monthly from the National Institute of Meteorology website (INMET, http://www.inmet.gov.br/portal), which is part of the Brazilian Government (INMET, 2023). The meteorological data were recorded through a meteorological station located in Belém-PA, which is 169.99 km away from the collection site in a straight line; the station is equipped with a Vaisala system, model MAWS 301 (Vaisala Corporation, Helsinki, Finland).

### 2.2 Essential oil extraction and yield calculation

The leaves were dried for 7 days in a climate-controlled environment and then pulverized. The leaves (50 g) were subjected to hydrodistillation (in duplicate) using a Clevenger apparatus (3 h), with the condensation system set at a temperature of 10°C–15°C. The essential oils obtained were centrifuged for 5 min at 3,000 rpm and dehydrated in anhydrous sodium sulfate (Na_2_SO_4_) under the same conditions (Maia and Andrade, 2009). The dry weights were used to calculate the oil yields. Essential oil yields were expressed as a percentage and calculated from the moisture-free biomass using the relationship between oil volume, plant sample mass, and moisture. The oils were stored in dark bottles for later chromatographic analysis (de Lima et al., 2024).

### 2.3 GC and GC-MS analyses

Gas chromatography–mass spectrometry (GC-MS) equipped with a gas chromatography–flame ionization detector (GC-FID) was used to analyze the composition of *A. oblanceolata* essential oils. A Shimadzu Model QP 2010 ultra-instrument (Shimadzu, Tokyo, Japan) equipped with an Rtx-5MS fused silica capillary column (30 m, 0.25 mm; 0.25 μm film thickness) as the stationary phase (Restek, Bellefonte, PA, United States) was used. Helium gas was used as the carrier gas, adjusted to 1.0 mL/min at 57.5 kPa. Oil samples were introduced into the instrument using split injection (ratio 1:20) of 1 μL of an n-hexane solution (5 μL of oil: 500 μL of n-hexane); the injector and interface temperatures were set to 250°C; the programmed oven temperature was 60°C–240°C (3°C/min), followed by 10-min isotherm. Electron ionization mass spectrometry (EIMS) was conducted at 70 eV, with an ion source temperature set to 200°C.

Mass spectra were obtained by automatic scanning, with fragment masses in the 35–400 *m/z* range. The mass spectra and retention indices of the samples were compared with those from the FFNSC-2 (Mondello, 2011) and Adams (Adams, 2017) commercial libraries. The retention indices of the volatile constituents were calculated using the linear equation by Van Den Dool and Kratz (1963), based on a homologous series of hydrocarbons (C8–C40, Sigma-Aldrich, St. Louis, MO, United States) under the same chromatographic conditions. GC-DIC analysis was performed on a Shimadzu QP-2010 instrument (Shimadzu, Tokyo, Japan) equipped with an FID under the same conditions described above, except that hydrogen was used as the carrier gas. The percentage composition of the oil sample was calculated from the GC-FID peak areas. Analyses were performed in triplicate.

### 2.4 Statistical analysis

PCA was applied to the essential oil components of *A. oblanceolata* leaves (>3.0%) (OriginPro/OriginLab 2024 Learning Edition Corporation, Northampton, MA, United States). HCA was performed considering Euclidean distance and Ward’s linkage. Statistical significance was assessed through a Tukey’s test (p < 0.05), and Pearson’s correlation coefficients (r) were calculated to determine the relationship between the analyzed climatic parameters (insolation, relative humidity, temperature, and precipitation) using GraphPad Prism software version 8.0.

## 3 Results and discussion

### 3.1 Relationship between essential oil yields and climatic parameters

**FIGURE 1:**
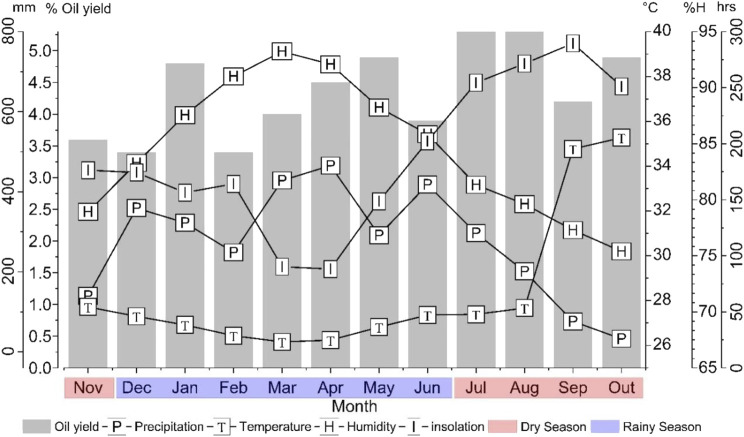
Relationship between the climatic parameters and the essential oil production of *Aloysia oblanceolata* during the seasonal variation study.

The climatic parameters of insolation, precipitation, temperature, and relative humidity were monitored from November 2022 to October 2023 to evaluate their influence on the production and composition of AoEO. Insolation values ranged from 88.2 h (April) to 289.5 h (September); monthly precipitation ranged from 32.7 mm (October) to 465.4 mm (April); temperature ranged from 26.7°C (May) to 35.8°C (October); and relative humidity from 75.4% (October) to 93.2% (March). The dry period in the region housing the collection site was November 2022 and July to October 2023, with an average precipitation of 149.3 ± 104.3 mm, and the rainy period was from December 2022 to June 2023, with an average precipitation of 361.9 ± 79.2 mm (Figure 1).

The Amazon holds approximately half of the Earth’s tropical rainforests and experiences torrential rains and droughts, varying in spatial and temporal scales. Therefore, the Amazon region has only two seasons: dry and rainy (Lean and Warrilow, 1989; Loureiro et al., 2014; Da Silva et al., 2019). With a humid and hot climate, the Amazon experiences the highest rainfall from December to April, the rainy season, and the lowest rainfall from June to November, the dry season. The remaining months are considered transition periods between seasons. However, these two seasons can vary from one year to the next, depending on the atmospheric phenomena that affect tropical regions (Hall et al., 1998; da Costa et al., 2020). In studies that evaluated the effect of seasonality on the composition and yields of essential oils, the climatic parameters obtained in 2022 indicated an atypical climate in the year studied (Barros et al., 2022; Santos et al., 2023).

**FIGURE 2:**
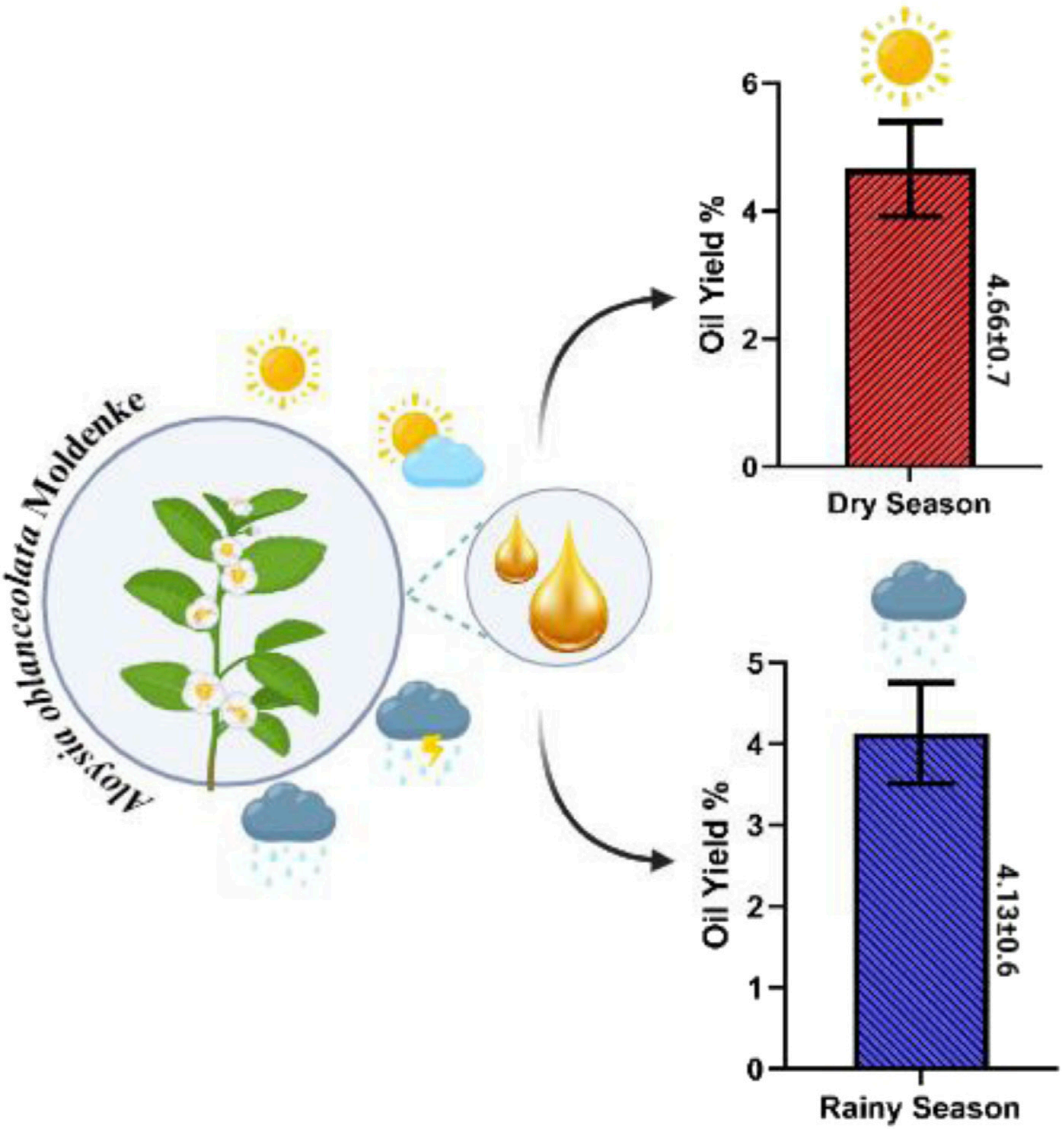
Essential oil yields from *Aloysia oblanceolata* in dry and rainy seasons.

In the seasonal study, AoEO yields ranged from 3.4% (December 2022 and February 2023) to 5.3% (August and September 2023), with an average of 4.3% ± 0.7% in the period studied (Figure 1). Essential oil yields did not show a significant difference (p > 0.05) between the dry (4.7% ± 0.7%) and rainy (4.1% ± 0.6%) periods (Figure 2).

**FIGURE 3:**
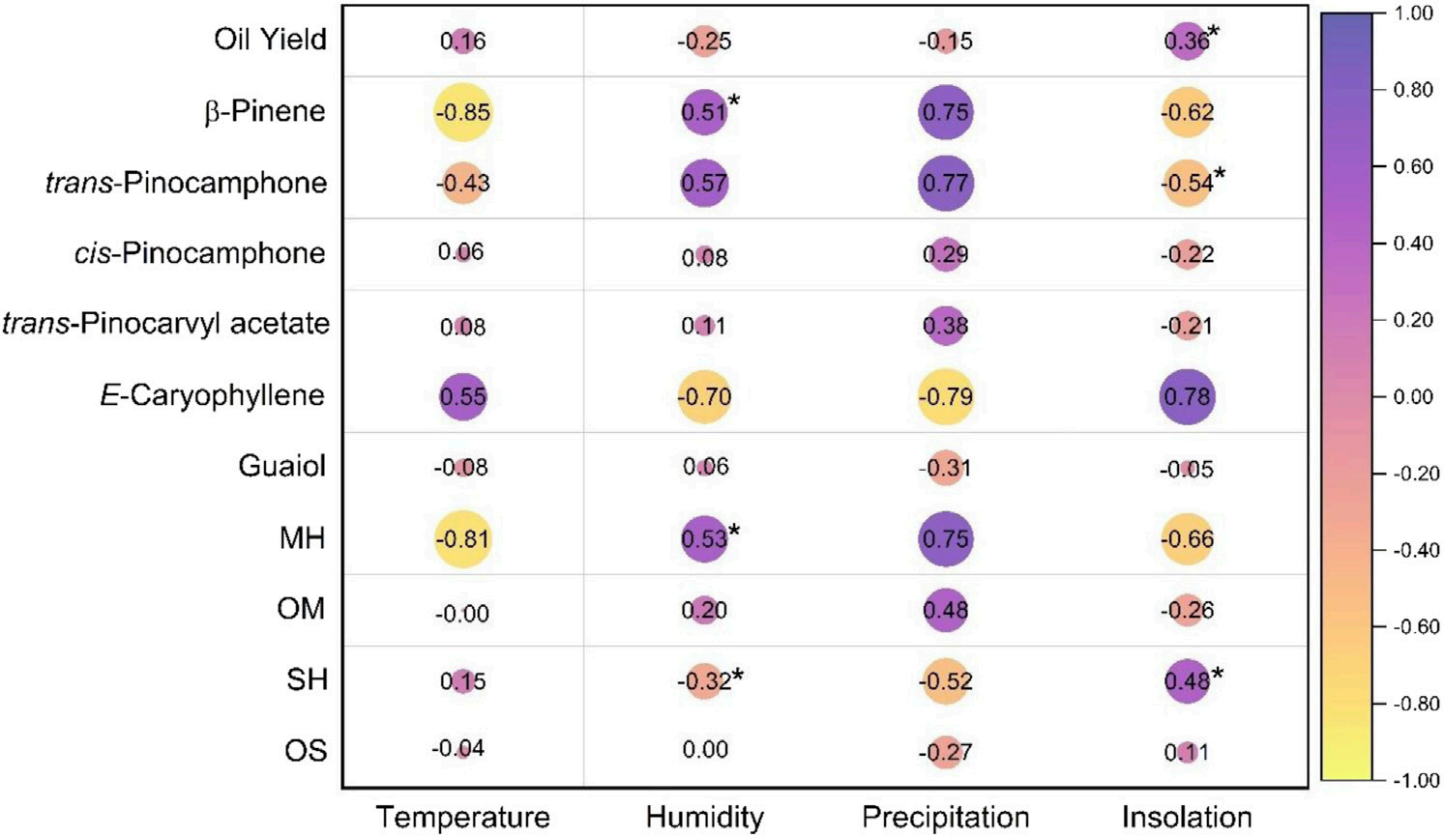
Correlation between the yields, main components, and classes of *Aloysia oblanceolata* oil compounds and climatic factors. MH, monoterpene hydrocarbon; OM, oxygenated monoterpene; SH, sesquiterpene hydrocarbon; OS, oxygenated sesquiterpene. *Significant correlation (p < 0.05).

Regarding the relationship between climatic parameters and essential oil yields, no significant correlations (p > 0.05) were observed with humidity (r = −0.25), sunlight (r = 0.36), precipitation (r = −0.15), or temperature (r = −0.16) (Figure 3).

A study that evaluated the effect of seasonality on the composition and yields of essential oils of *Lippia alba* leaves, collected in Belém, Pará, Brazil, did not present a significant difference in oil yields between the dry period (1.1% ± 0.3%) and the rainy period (1.7% ± 0.5%) (Barros et al., 2022). On the other hand, during the evaluation of the effect of seasonality on the composition and yields of essential oils of *Aloysia triphylla* leaves, cultivated and collected in Rio Grande do Sul, Brazil, oil yields were different during the summer (0.42%), autumn (0.31%), winter (0.19%), and spring (0.30%) seasons. The season with the lowest rainfall presented the best yield during the studied period (Parodi et al., 2020).

### 3.2 Seasonal effects on *Aloysia oblanceolata* oil composition

**FIGURE 4:**
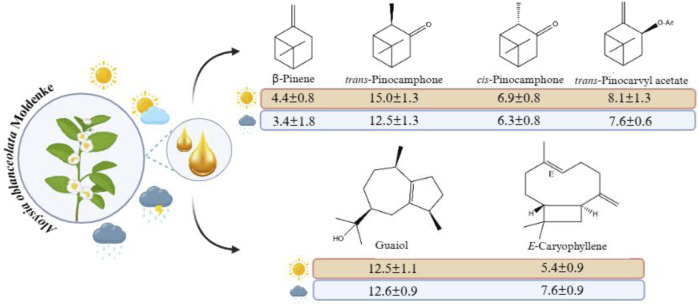
Chemical structures of the main constituents identified in *Aloysia oblanceolata* oils during the seasonal period.

The essential oils from *Aloysia oblanceolata* contain 38 chemical constituents (Table 1). These constituents comprise 94.6%–97.1% of the oils analyzed in the seasonal study over the 12-month period. The predominant compounds in the essential oils of the leaf samples were oxygenated monoterpenes (33.2%–48.1%), oxygenated sesquiterpenes (18.5%–27.8%), sesquiterpene hydrocarbons (19.0%–27.3%), and monoterpene hydrocarbons (2.4%–10.7%). The main constituents identified in the essential oils of the leaves during the seasonal study were *trans*-pinocamphone (11.1%–16.4%), guaiol (10.8%–14.2%), *trans*-pinocarvyl acetate (6.1%–9.5%), *cis*-pinocamphone (5.7%–8.4%), and β-pinene (0.9%–5.8%). The structures of the constituents are shown in Figure 4.

**TABLE 1:** Yields and chemical composition of essential oils from *Aloysia oblanceolata* leaves relative to the seasonal variation study.

No	RI_(C)_	RI_(L)_	Period	Dry	Rainy							Dry			
			Collection months	Nov	Dec	Jan	Feb	Mar	Apr	May	Jun	Jul	Aug	Sep	Oct
			Oil yield	3.6	3.4	4.8	3.4	4.0	4.5	4.9	3.9	5.3	5.3	4.2	4.9
			Constituent	(%)^c^											
1	934	932^a^	α-Pinene	0.6	0.6	0.3	0.4	0.5	0.6	0.4	0.5	0.5	0.4	0.6	1.1
2	974	969^a^	Sabinene	0.2	0.3	0.2	0.2	0.2	0.2	0.2	0.3	0.2	0.2		
**3**	**980**	**974** ^a^	**β-Pinene**	**5.3**	**5.8**	**3.9**	**4.0**	**5.2**	**5.1**	**4.1**	**5.5**	**4.3**	**4.1**	**0.9**	**2.2**
4	992	988^a^	Myrcene	1.0	1.2	0.8	0.8	1.0	0.9	0.8	1.1	0.8	0.7	0.2	0.4
5	1,029	1,024^a^	Limonene	1.6	1.8	1.4	1.3	1.7	1.6	1.3	1.7	1.3	1.3	0.4	0.8
6	1,047	1,044^a^	*E*-β-ocimene	0.6	0.8	0.6	0.7	0.8	0.6	0.6	0.8	0.4	0.3	0.1	0.4
7	1,089	1,086^a^	Terpinolene	0.2	0.2	0.1	0.2	0.2	0.2	0.1	0.2	0.1	0.1	0.2	
8	1,101	1,095^a^	Linalool	1.7	2.5	1.7	1.7	2.3	2.3	1.8	2.3	1.7	1.8	1.7	2.2
9	1,126	1,122^a^	α-Campholenal	0.2	0.3		0.2	0.2	0.2	0.2	0.2	0.2	0.1	0.2	0.3
10	1,140	1,135^a^	*trans*-Pinocarveol	2.5	3.5	4.6	2.8	3.2	3.3	2.9	3.2	2.8	3.6	3.3	3.4
**11**	**1,163**	**1,158** ^a^	***trans*-Pinocamphone**	**11.1**	**16.4**	**14.9**	**13.5**	**16.0**	**15.4**	**12.9**	**15.9**	**13.1**	**13.2**	**11.0**	**13.9**
12	1165	1,160^a^	Pinocarvone	1.7	2.3	1.9	1.9	2.2	2.2	2.1	2.4	2.1	2.1	2.0	2.3
**13**	**1,176**	**1,172** ^a^	***cis*-Pinocamphone**	**5.7**	**8.4**	**7.0**	**6.2**	**7.1**	**6.9**	**5.9**	**7.1**	**6.0**	**6.2**	**5.8**	**7.8**
14	1178	1,174^a^	Terpinen-4-ol	0.3	0.4	0.4	0.3	0.3	0.3	0.3	0.3	0.4	0.3	0.4	0.6
15	1,191	1,186^a^	α-Terpineol	0.2	0.3	0.3	0.2	0.2	0.3	0.2	0.2	0.2	0.2	0.2	0.3
16	1,198	1,195^a^	Myrtenol	2.0	3.1	1.9	1.8	2.4	1.8	2.2	2.4	2.5	2.5	3.6	3.2
17	1,204	1,199^a^	γ-Terpineol	0.2	0.3	0.3	0.2		0.2			0.1		0.2	0.2
18	1,219	1,215^a^	*trans*-Carveol	0.2	0.4	0.2	0.2	0.3	0.3	0.2	0.2	0.3		0.3	0.3
19	1,286	1,287^a^	Bornyl acetate	0.5	0.7	0.5	0.5	0.6	0.6	0.5	0.6	0.5	0.5	0.6	0.6
**20**	**1,302**	**1,311** ^a^	***trans*-Pinocarvyl acetate**	**6.9**	**9.5**	**6.1**	**6.9**	**8.9**	**9.1**	**7.4**	**8.9**	**7.6**	**7.2**	**7.8**	**8.6**
21	1,338	1,335^a^	δ-Elemene	0.2	0.1	0.2	0.2		0.1	0.2		0.2	0.1	0.2	0,3
22	1,385	1,387^a^	β-Bourbonene	0.1		0.3	0.2		0.1	0.2		0.3	0.3	0.3	
23	1,393	1,389^a^	β-Elemene	1.1	0.9	1.2	1.1	0.9	0.9		0.9	1.2	1.1	1.2	0.9
**24**	**1,422**	**1,417** ^a^	***E*-Caryophyllene**	**7.1**	**4.8**	**6.3**	**6.0**	**4.8**	**4.6**	**6.6**	**4.7**	**7.5**	**7.7**	**9.0**	**6.7**
25	1,430	1,430^a^	β-Copaene		0.1			0.2					0.1		
26	1,435	1,434^a^	γ-Elemene	2.1	1.5	2.3	2.1	1.7	1.5	2.2	1.6	2.1	1.8	2.3	1.5
27	1,455	1,452^a^	α-Humulene	1.9	1.3	1.7	1.6	1.3	1.3	1.9	1.3	2.0	2.0	2.2	1.6
28	1,462	1,464^a^	9-epi-*E*-caryophyllene	0.2	0.1	0.3	0.2	0.1	0.2	0.3	0.1	0.3	0.2	0.2	
29	1,483	1,478^a^	γ-Muurolene	5.3	4.2	6.4	6.1	5.2	4.6	6.9	5.2	6.0	5.2	3.8	3.9
30	1,498	1,500^a^	Bicyclogermacrene	2.5	1.8	2.4	2.4	1.8	1.6	2.4	1.8	2.2	2.0	2.8	2.3
31	1,516	1,522^a^	δ-Cadinene	0.3	0.3	0.3	0.3	0.3	0.4	0.3	0.3	0.3	0.4	0.3	0.2
32	1,551	1,548^a^	Elemol	2.2	1.6	1.6	2.3	2.3	1.9	1.9	2.2	1.6	1.7	1.6	2.1
33	1,559	1,559^a^	Germacrene B	3.2	2.3	3.5	3.2	2.6	2.4	3.3	2.6	3.3	2.9	3.4	2.4
34	1,580	1,577^a^	Spathulenol	1.4	0.8	0.9	1.0	0.8	1.7	1.2	0.9	1.6	2.1	3.2	2.4
35	1,585	1,582^a^	Caryophyllene oxide	3.5	2.6	2.9	3.1	2.2	3.0	2.9	2.2	3.4	3.9	5.4	4.6
**36**	**1,603**	**1,600** ^a^	**Guaiol**	**14.2**	**10.8**	**13.4**	**14.0**	**12.3**	**11.4**	**13.1**	**12.4**	**12.4**	**11.8**	**12.6**	**12.1**
37	1,611	1,609^b^	Rosifoliol	1.5		1.4	1.4	1.3	1.4	1.4	1.1	1.5	1.6	1.8	1.6
38	1,671	1,670^a^	Bulnesol	6.6	4.3	4.9	6.3	6.0	5.4	6.2	6.0	5.0	5.0	4.8	4.9
Monoterpene hydrocarbons				9.5	10.7	7.3	7.6	9.6	9.2	7.5	10.1	7.6	7.1	2.4	4.9
Oxygenated monoterpenes				33.2	48.1	39.8	36.4	43.7	42.9	36.6	43.7	37.5	37.7	37.1	43.7
Sesquiterpene hydrocarbons				26.2	19.0	26.5	25.7	21.2	19.6	26.2	20.7	27.0	25.5	27.3	21.6
Oxygenated sesquiterpenes				27.2	18.5	23.5	25.8	22.6	22.9	24.8	22.6	23.9	24.4	27.8	25.6
**Total (%)**				**96.1**	**96.3**	**97.1**	**95.5**	**97.1**	**94.6**	**95.1**	**97.1**	**96.0**	**94.7**	**94.6**	**95.8**

**RI**_**(C)**_ = calculated retention index; **RI**_**(L)**_ = literature retention index.

^a^ Adams (2007).

^b^ Mondello (2011);

^c^ The standard deviation was less than 2.0 (n = 2). The bold represents main constituents.

The chemical constituents that demonstrated a significant correlation with climatic factors were *trans*-pinocamphone, which presented a moderate negative correlation with insolation (r = −0.54), and β-pinene, which presented a positive moderate correlation with humidity (0.51). However, some constituents had no significant correlation with climate parameters (p < 0.05); for example, *trans*-pinocamphone presented a moderate positive correlation with humidity (r = 0.57) and a strong positive correlation with precipitation (r = 0.77); β-pinene presented a strong negative correlation with temperature (r = −0.85), a moderate negative correlation with insolation (r = −0.62), and a strong correlation with precipitation (r = 0.75); *E*-caryophyllene presented a moderate correlation with temperature (r = 0.55) and strong correlations with insolation (r = 0.78), precipitation (r = −0.79), and humidity (r = 0.70). Thus, the monoterpene hydrocarbon class showed a strong negative correlation with temperature (r = −0.81), a moderate negative correlation with insolation (r = −0.66), a moderate positive correlation with humidity (r = 0.53, with significance), and a strong correlation with precipitation (r = 0.75), while the sesquiterpene hydrocarbon class showed a moderate negative correlation with precipitation (r = −0.52) and a moderate positive correlation with insolation (r = 0.48) (Figure 3).

During the seasonal study, variations in the amounts of the main constituents were observed throughout the seasons. In the rainy season, *trans*-pinocamphone presented the highest contents of 15.0%, while in the dry season, the content was reduced to 12.5%. On the other hand, guaiol displayed a concentration of 10.8% in the rainy season, which increased to 12.4% in the dry season. *trans*-Pinocarvyl acetate presented contents of 8.1% in the rainy season and 7.6% in the dry season. *cis*-Pinocamphone content varied from 6.9% in the rainy season to 6.3% in the dry season. *E*-caryophyllene contents increased from 5.4% in the rainy season to 7.6% in the dry season. In contrast, β-pinene recorded the highest level in the rainy season (4.8%), while in the dry season, a lower level was observed (3.4%).

There is still limited information available regarding the mechanisms behind the influence of environmental factors on terpene emissions from plants. Studies show that temperature, vapor pressure of the terpenes, the humidity of the air surrounding the leaf, and the exposure area of essential oils are all involved in the passive release of constitutive terpenes, in a manner that is often independent of the stomatal opening. Furthermore, monoterpene emission can also be influenced by thermal stress/heat stress, when plants are exposed to a high temperature that affects some physiological processes. Due to heat stress, stomata open, and monoterpenes are likely to be released into the atmosphere immediately after their synthesis from non-storage tissues (Malik et al., 2023).

### 3.3 Multivariate analysis of the essential oils from *Aloysia oblanceolata*

**FIGURE 5:**
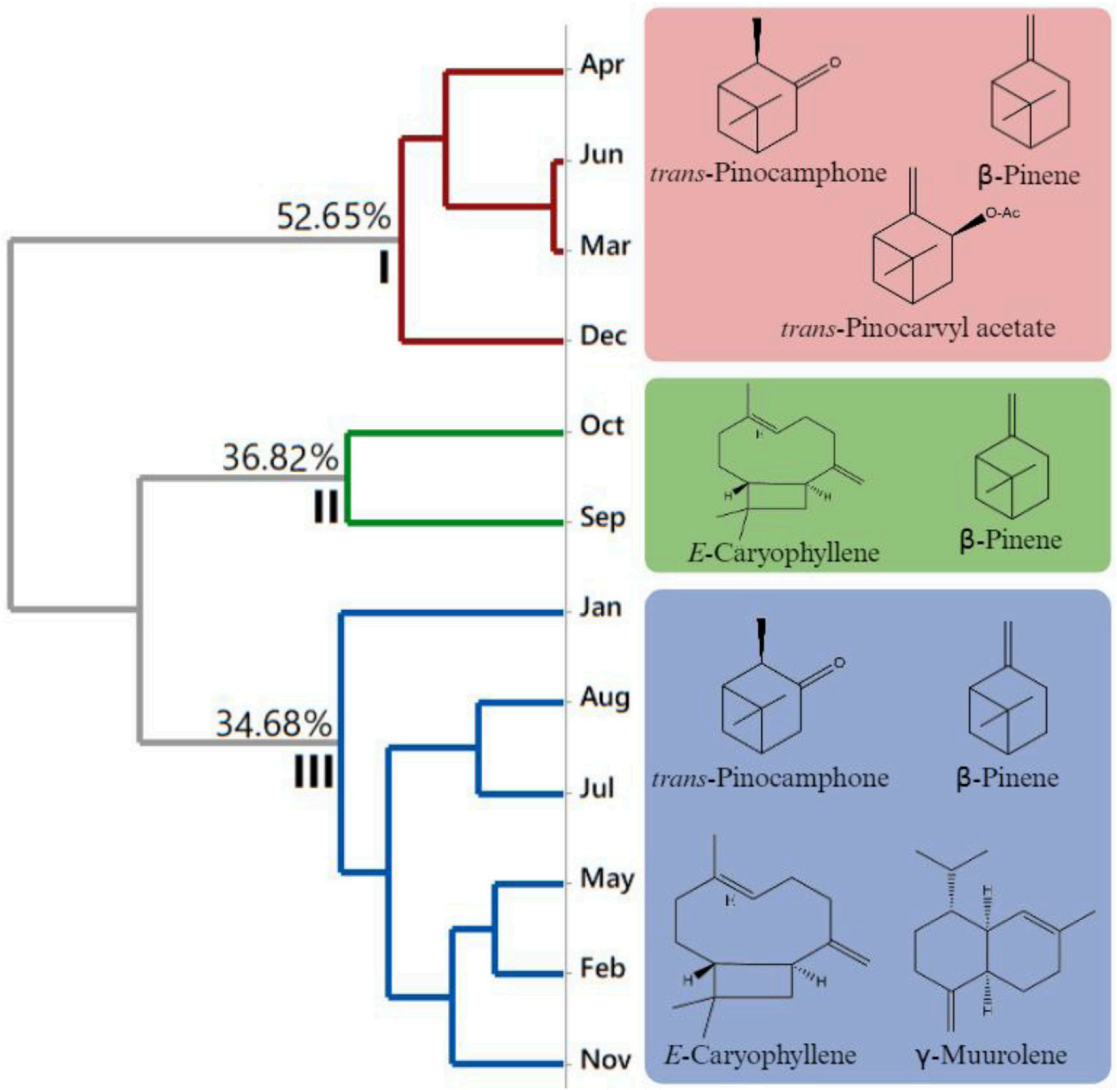
Hierarchical cluster analysis of the main constituents of the essential oils from *Aloysia oblanceolata.*

HCA and PCA were plotted, with the main constituents of the essential oils showing values above 3%. Applying HCA provided the dendrogram shown in Figure 5, which presents the formation of three groups of *Aloysia oblanceolata*. Group I was composed of oils extracted from April, June, March, and December, with a similarity of 52.65%. Group II presented a similarity of 36.82%, represented by the samples extracted from October and September. Group III presented a similarity of 34.68%, which was represented by the samples extracted from January, August, July, May, February, and November.

In the seasonal variation analysis of the chemical composition of the essential oils, variations in the concentration of the main constituents were observed throughout the dry and rainy periods. *trans*-Pinocarvyl acetate was the predominant component of group I (9.1% ± 0.3%), while *E*-caryophyllene was the main constituent of group II (7.8% ± 1.6%), and *trans*-Pinocamphone (13.1% ± 1.2%) in group III.

**FIGURE 6:**
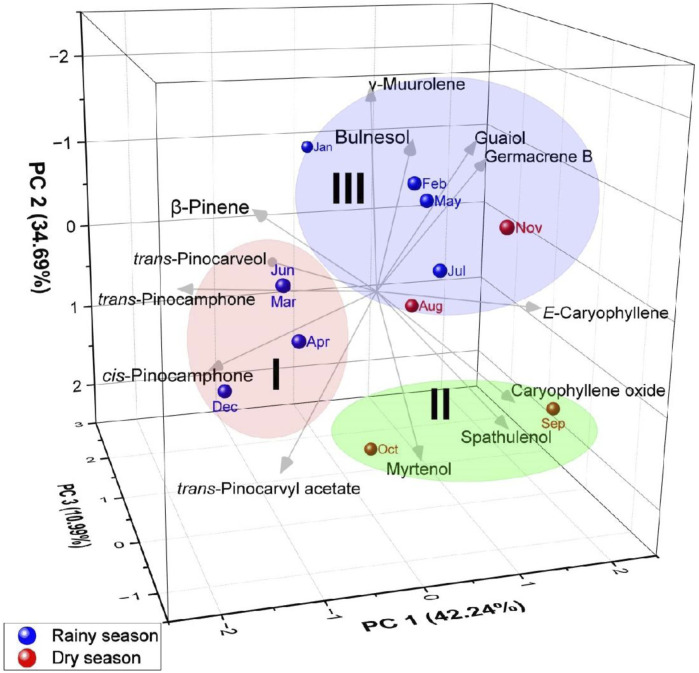
Principal component analysis of the main constituents of the essential oils from *Aloysia oblanceolata*.

These results indicate seasonal variations in the chemical composition of the essential oils, possibly influenced by environmental factors and plant development. PCA (Figure 6) of the constituents of the essential oils of *Aloysia oblanceolata* elucidated 87.92% of the data variability. Like HCA, PCA confirmed the formation of three distinct groups.

**FIGURE 7:**
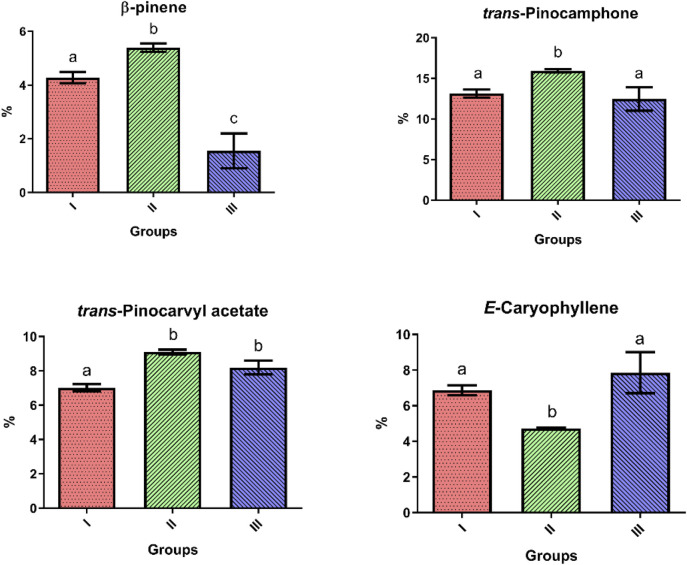
Analysis of the standard deviations of the three groups of *Aloysia oblanceolata* from the seasonal variation study. Mean ± standard deviation. Values with the same letters on the bars are considered not statistically different in Tukey’s test (p > 0.05).

The analysis of the mean content and standard deviation of the constituents present in the AoEO (Figure 7) showed that Group I had statistical differences (Tukey’s test, p < 0.05) when compared to Groups II and III in terms of *trans*-pinocarvyl acetate contents (I = 7.0 ± 0.5%; II = 9.1 ± 0.3%; III = 8.2 ± 0.6%). Group II differed from the other groups in terms of β-pinene contents (I = 4.3 ± 0.5%; II = 5.2 ± 0.3%; III = 1.6 ± 0.9%), *trans*-pinocamphone (I = 13.1 ± 1.2%; II = 15.9 ± 0.4%; III = 12.5 ± 2.1%), and *E*-caryophyllene contents (I = 6.9 ± 0.7%; II = 4.7 ± 0.1%; III = 7.9 ± 1.6%). Furthermore, Group III differed from the other groups in terms of β-pinene contents (I = 4.3 ± 0.5%; II = 5.2 ± 0.3%; III = 1.6 ± 0.9%).

**FIGURE 8:**
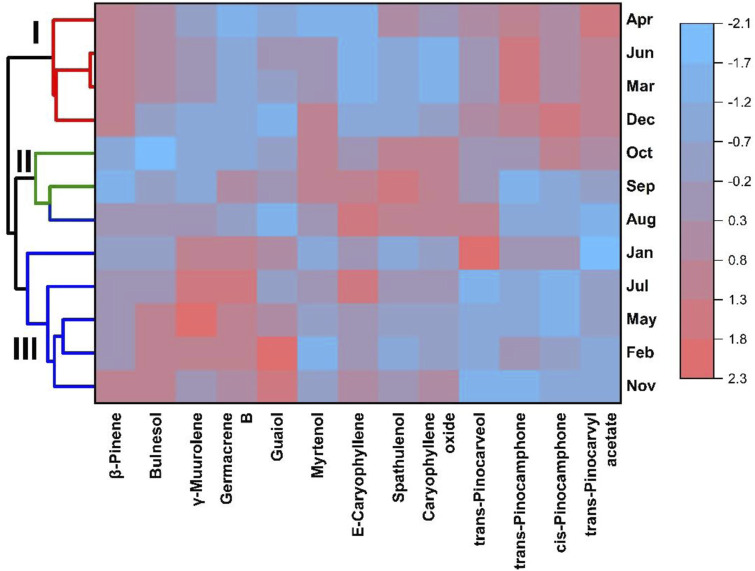
Clustering heatmap with the constituents of the essential oils from *Aloysia oblanceolata* samples.

Applying a multivariate analysis combining a heatmap with hierarchical clustering analysis (Figure 8), with the chemical constituents revealed a color pattern that varied with a gradual increase in intensity, indicating the lowest to the highest degree. The clustered heatmap (Figure 8) confirmed the clustering results obtained in PCA and HCA (see Figures 5, 6).

## 4 Conclusion

The essential oils from *Aloysia oblanceolate,* found in the Amazon region, has a chemical composition that is rich in *trans*-pinocamphone, β-pinene, *E*-caryophyllene, and *trans*-pinocarvyl acetate, which demonstrated correlation with climatic factors, evidencing that environmental factors and plant development influence the chemical composition of the essential oils.

It is vital to consider the effects of climatic conditions in the production and use of the essential oils from *A. oblanceolata* , especially in traditional medicine. The efficacy may vary depending on the oil’s chemical composition. This study expands scientific knowledge about *A. oblanceolata* leaf essential oils and provides valuable information for a more sustainable use of this species.

## Data Availability

The original contributions presented in the study are included in the article/supplementary material; further inquiries can be directed to the corresponding author.

## Abbreviations

HCA, hierarchical cluster analysis; PCA, principal component analysis; GC-MS, gas chromatography–mass spectrometry; GC-FID, gas chromatography–flame ionization detection; AoEO, *Aloysia oblanceolata* essential oil.

## References

[B1] Adams R. P. (2007). Identification of essential oil components by gás chromatography/massspectrometry. Carol Stream, IL. Allured Publishing Corporation.

[B2] Adams R. P. (2017). Identification of essential oil components by gas chromatography/mass spectrometry. Carol Stream, IL. Allured Publishing Corporation.

[B3] Alijar Souza M. Petry F. Vidor Morgan L. Dal Magro J. Müller L. G. (2022). Biological properties of *Aloysia gratissima* (gillies and hook.) tronc. (Verbenaceae). Evidence-Based Complement. Altern. Med. 2022, 1–8. 10.1155/2022/1119435 PMC8791711 35096101

[B4] Barros L. de S. P. Santos da Cruz E. de N. de Araújo Guimarães B. Setzer W. N. Veras Mourão R. H. do Rosário da Silva J. K. (2022). Chemometric analysis of the seasonal variation in the essential oil composition and antioxidant activity of a new geraniol chemotype of *Lippia alba* (Mill.) N.E.Br. ex Britton and P. Wilson from the Brazilian Amazon. Biochem. Syst. Ecol. 105, 104503. 10.1016/j.bse.2022.104503

[B5] Benovit S. C. Silva L. L. Salbego J. Loro V. L. Mallmann C. A. Baldisserotto B. (2015). Anesthetic activity and bio-guided fractionation of the essential oil of *Aloysia gratissima* (Gillies and Hook.) Tronc. in silver catfish *Rhamdia quelen* . An. Acad. Bras. Cienc. 87, 1675–1689. 10.1590/0001-3765201520140223 26221984

[B6] da Costa J. S. Barroso A. S. Mourão R. H. V. da Silva J. K. R. Maia J. G. S. Figueiredo P. L. B. (2020). Seasonal and antioxidant evaluation of essential oil from *Eugenia uniflora* L., curzerene-rich, thermally produced *in situ* . Biomolecules 10, 328. 10.3390/biom10020328 32092893 PMC7072495

[B7] Da Silva P. E. Santos e Silva C. M. Spyrides M. H. C. Andrade L. de M. B. (2019). Precipitation and air temperature extremes in the Amazon and northeast Brazil. Int. J. Climatol. 39, 579–595. 10.1002/joc.5829

[B8] de Cássia da Silveira e Sá R. Andrade L. dos Reis Barreto de Oliveira R. de Sousa D. (2014). A review on anti-inflammatory activity of phenylpropanoids found in essential oils. Molecules 19, 1459–1480. 10.3390/molecules19021459 24473208 PMC6270723

[B9] de Lima M. N. N. Santos P. V. L. Jerônimo L. B. Viana R. M. da Silva J. K. Setzer W. N. (2024). Seasonal influence on the essential oil chemical composition of *Hyptis crenata* Pohl ex Benth.: a valuable plant from Marajó, Brazil. Front. Chem. 12, 1397634. 10.3389/fchem.2024.1397634 38863674 PMC11165112

[B10] Gobbo-Neto L. Lopes N. P. (2007). Plantas medicinais: fatores de influência no conteúdo de metabólitos secundários. Quim. Nova 30, 374–381. 10.1590/S0100-40422007000200026

[B11] Gressler L. T. Riffel A. P. K. Parodi T. V. Saccol E. M. H. Koakoski G. da Costa S. T. (2014). Silver catfish *Rhamdia quelen* immersion anaesthesia with essential oil of *Aloysia triphylla* (L’Hérit) Britton or tricaine methanesulfonate: effect on stress response and antioxidant status. Aquac. Res. 45, 1061–1072. 10.1111/are.12043

[B12] Hall J. C. Heel K. A. Papadimitriou J. M. Platell C. (1998). The pathobiology of peritonitis. Gastroenterology 114, 185–196. 10.1016/S0016-5085(98)70646-8 9428232

[B13] Jerônimo L. B. de Araújo J. A. C. da Silva J. K. R. Mourão R. H. V. Setzer W. N. Figueiredo P. L. B. (2024). Seasonality’s effects on the chemical composition and antiradical capacity of the floral essential oil of *Acmella oleracea* (L.) R.K. Jansen cultivated in the Brazilian Amazon. Horticulturae 10, 925. 10.3390/horticulturae10090925

[B14] Lean J. Warrilow D. A. (1989). Simulation of the regional climatic impact of Amazon deforestation. Nature 342, 411–413. 10.1038/342411a0

[B15] Loureiro R. S. Saraiva J. M. Saraiva I. Senna R. C. Fredó A. S. (2014). Estudo dos eventos extremos de precipitação ocorridos em 2009 no estado do Pará. Rev. Bras. Meteorol. 29, 83–94. 10.1590/0102-778620130054

[B16] Maia J. G. S. Andrade E. H. A. (2009). Database of the Amazon aromatic plants and their essential oils. Quim. Nova 32, 595–622. 10.1590/S0100-40422009000300006

[B17] Malik T. G. Sahu L. K. Gupta M. Mir B. A. Gajbhiye T. Dubey R. (2023). Environmental factors affecting monoterpene emissions from terrestrial vegetation. Plants 12, 3146. 10.3390/plants12173146 37687392 PMC10489858

[B18] Marx H. E. O’Leary N. Yuan Y. Lu‐Irving P. Tank D. C. Múlgura M. E. (2010). A molecular phylogeny and classification of Verbenaceae. Am. J. Bot. 97, 1647–1663. 10.3732/ajb.1000144 21616800

[B19] Mohammadhosseini M. Frezza C. Venditti A. (2022). An overview of the genus *Aloysia Paláu* (Verbenaceae): essential oil composition, ethnobotany and biological activities. Nat. Prod. Res. 0, 1–17. 10.1080/14786419.2021.1907576 33843369

[B20] Mondello L. (2011). FFNSC 2: flavors and fragrances of natural and synthetic compounds, mass spectral database. Hoboken, NJ: John Wiley and Sons Inc.

[B21] O’Leary N. Lu-Irving P. Moroni P. Siedo S. (2016a). Taxonomic revision of *Aloysia* (verbenaceae, Lantaneae) in South America. Ann. Mo. Bot. Gard. 101, 568–609. 10.3417/2013015

[B22] Parodi T. V. Gressler L. T. Silva L. de L. Becker A. G. Schmidt D. Caron B. O. (2020). Chemical composition of the essential oil of *Aloysia triphylla* under seasonal influence and its anaesthetic activity in fish. Aquac. Res. 51, 2515–2524. 10.1111/are.14594

[B23] Romero M. D. María E. Alicia D. De M. Tribe V. I. Martínez S. (2002). Morfología de las inflorescencias en verbenaceae, verbenoideae iii: * tribu Lantanea. Darwiniana 40, 1–15.

[B24] Santos P. V. L. da Cruz E. de N. S. Nunes J. de A. Mourão R. H. V. do Nascimento W. M. O. Maia J. G. S. (2023). Seasonal influence on volatile composition of *Psidium friedrichsthalianum* leaves, sampled in the Brazilian Amazon. Horticulturae 9, 768. 10.3390/horticulturae9070768

[B25] Souza A. A. Wiest J. M. (2007). Atividade anti-bacteriana de *Aloysia gratissima* (Gill et Hook) Tronc. (garupá, ervasanta), usada na medicina tradicional no Rio Grande do Sul - Brasil. Rev. Bras. Plantas Med. 9, 23–29.

[B26] Ud-Daula A. F. M. S. Demirci F. Abu Salim K. Demirci B. Lim L. B. L. Baser K. H. C. (2016). Chemical composition, antioxidant and antimicrobial activities of essential oils from leaves, aerial stems, basal stems, and rhizomes of *Etlingera fimbriobracteata* (K.Schum.) R.M.Sm. Ind. Crops Prod. 84, 189–198. 10.1016/j.indcrop.2015.12.034

[B27] Van Den Dool H. Kratz P. D. (1963). A generalization of the retention index system including linear temperature programmed gas—liquid partition chromatography. J. Chromatogr. A 11, 463–471. 10.1016/s0021-9673(01)80947-x 14062605

[B28] Zeni A. L. B. Zomkowski A. D. E. Dal-Cim T. Maraschin M. Rodrigues A. L. S. Tasca C. I. (2011). Antidepressant-like and neuroprotective effects of *Aloysia gratissima*: investigation of involvement of l-arginine-nitric oxide-cyclic guanosine monophosphate pathway. J. Ethnopharmacol. 137, 864–874. 10.1016/j.jep.2011.07.009 21767626

